# Combined Percutaneous Tracheostomy and Endoscopic Gastrostomy Tubes in COVID-19: A Prospective Series of Patient Outcomes

**DOI:** 10.1177/08850666211038875

**Published:** 2021-08-23

**Authors:** Catherine L. Oberg, Colleen Keyes, Tanmay S. Panchabhai, Muhammed Sajawal Ali, Scott S. Oh, Tristan R. Grogan, James Mojica, Hugh Auchincloss, Natalie Pulido, Kelsey Brait, Erik E. Folch

**Affiliations:** 1 2348 Massachusetts General Hospital, Harvard Medical School, Boston, MA, USA; 2 12222 David Geffen School of Medicine at UCLA, Los Angeles, CA, USA; 3 114516 University Hospitals Cleveland Medical Center, Cleveland, OH, USA; 4 1259 University of Michigan, Ann Arbor, MI, USA

**Keywords:** mechanical ventilation, respiratory failure, tracheostomy tube, gastrostomy, COVID-19

## Abstract

**Background:** A significant number of patients with severe respiratory failure related to COVID-19 require prolonged mechanical ventilation. Minimal data exists regarding the timing, safety, and efficacy of combined bedside percutaneous tracheostomy and endoscopy gastrostomy tube placement in these patients. The safety for healthcare providers is also in question. This study's objective was to evaluate the effectiveness and safety of combined bedside tracheostomy and gastrostomy tube placement in COVID-19 patients. **Design and Methods:** This is a single arm, prospective cohort study in patients with COVID-19 and acute respiratory failure requiring prolonged mechanical ventilation who underwent bedside tracheostomy and percutaneous endoscopic gastrostomy placement. Detailed clinical and procedural data were collected. Descriptive statistics were employed and time to event curves were estimated and plotted using the Kaplan Meier method for clinically relevant prespecified endpoints. **Results:** Among 58 patients, the median total intensive care unit (ICU) length of stay was 29 days (24.7-33.3) with a median of 10 days (6.3-13.7) postprocedure. Nearly 88% of patients were weaned from mechanical ventilation postprocedure at a median of 9 days (6-12); 94% of these were decannulated. Sixty-day mortality was 10.3%. Almost 90% of patients were discharged alive from the hospital. All procedures were done at bedside with no patient transfer required out of the ICU. A median of 3.0 healthcare personnel total were present in the room per procedure. **Conclusion:** This study shows that survival of critically ill COVID-19 patients after tracheostomy and gastrostomy was nearly 90%. The time-to-event curves are encouraging regarding time to weaning, downsizing, decannulation, and discharge. A combined procedure minimizes the risk of virus transmission to healthcare providers in addition to decreasing the number of anesthetic episodes, transfusions, and transfers patients must undergo. This approach should be considered in critically ill COVID-19 patients requiring prolonged mechanical ventilation.

## Introduction

The coronavirus disease 2019 (COVID-19) pandemic caused by the SARS-CoV-2 virus has taken the world by storm, infecting over 179 million patients globally as of June 23, 2021. In the United States (US) alone, over 33.5 million cases have been reported.^1^ This highly contagious respiratory virus typically causes mild symptoms such as fever, cough, fatigue, and dyspnea, however a subset of patients will develop critical illness, often including acute respiratory failure, acute respiratory distress syndrome (ARDS), and multiorgan failure.^2^ In a retrospective review of 191 patients hospitalized with COVID-19 in Wuhan, China, 32 (16.7%) required invasive mechanical ventilation with a mean initiation at 14.5 days from symptom onset.^3^ A meta-analysis of 3062 patients with COVID-19 across 38 studies showed an incidence of ARDS in 19.5%.^4^ Of 73 ventilated patients with COVID-19 and ARDS in Milan, Italy, 45.2% continued to need invasive mechanical ventilation at day 19 of their intensive care unit (ICU) course.^5^ In 21 critically ill COVID-19 patients in Washington, United States, 71% patients required mechanical ventilation and 38% of those were unable to be weaned by day 25.^6^

These data suggest that a significant number of patients admitted to the ICU with severe respiratory failure will require prolonged mechanical ventilation. The pressing question is how to provide this support in the most efficient, safe, and effective fashion while minimizing the risk of virus transmission to healthcare personnel (HCP). Additionally, resource utilization has become paramount as supply chains have been disrupted throughout the pandemic. Shortages of personal protective equipment (PPE) in addition to other critical medical supplies have been reported throughout the United States and the world.^7^ Efforts to minimize waste of healthcare resources and maximize benefits are also of the utmost importance.

Tracheostomy placement is used in patients with prolonged respiratory failure for many reasons—it improves patient communication, work of breathing, need for sedation, and overall patient comfort.^8,9^ It also allows for improved oral hygiene and decreased injury to the vocal cords and glottis as the endotracheal tube (ETT) can be removed.^10^ Finally, it may facilitate weaning from mechanical ventilation as it decreases airway resistance, improves secretion clearance, decreases aspiration due to better glottic function, and allows for transfer out of the ICU.^11^ These tubes can be placed either percutaneously or surgically, with most centers deferring to their available human capital and subsequently preferred technique. The percutaneous approach has been shown to require less procedural time, has less wound infection and scarring, and is less expensive than the surgical technique. No difference in mortality or intraprocedural complication rates has been shown.^12,13^ The percutaneous technique is typically performed at bedside in the ICU while surgical placement is usually done in the operating room (OR). Gastrostomy tubes are often required in these patients as well to provide nutritional support, particularly in patients who are not able to be weaned from mechanical ventilation.^14^ If longer term support is needed, gastrostomy tubes are frequently preferred over nasogastric or orogastric tubes for stability as well as to mitigate the risk of sinusitis, pharyngeal discomfort, epistaxis, and short-term aspiration.^15,16^ Gastrostomy tubes are placed by a wide array of physicians, including gastroenterologists, general surgeons, interventional pulmonologists, and interventional radiologists, and utilize various techniques, such as endoscopic, radiologic, or surgical placement.^17^ Endoscopic placement can be done at bedside in the ICU while surgical or radiologic placement is typically performed in the OR or procedural suite.

A significant portion of patients with COVID-19 associated respiratory failure who have survived the acute phase of disease are now requiring prolonged care. The exact timing and technique for tracheostomy placement is unclear, with minimal evidence-based guidelines available. Several societal guidelines and expert opinion papers recommend tracheostomy placement after day 10 to 14 of mechanical ventilation.^18–20^ The choice of procedure type and location are generally left to the discretion of the provider with priorities placed on utilizing the most experienced operator while balancing the risks to both provider and patient. Similar questions exist regarding optimal timing and technique for gastrostomy tube placement. As these procedures are high risk for aerosol generation, the goal should be to perform them in a safe and effective manner while minimizing risk to HCP involved. This can involve steps such as minimizing the number of personnel in the room at the time of placement, utilizing experts in each procedure in order to decrease overall procedural length, performing these procedures at bedside in the ICU to decrease the number of patient transfers, and performing both procedures during one anesthetic episode.

In this study, we prospectively evaluated ICU patients diagnosed with SARS-CoV-2 undergoing percutaneous tracheostomy and gastrostomy tube placements by the Interventional Pulmonology service at the Massachusetts General Hospital in order to assess the impact of these procedures on patients with COVID-19. Included in this analysis are comprehensive details of patient demographics, comorbid conditions, and ICU-specific data, such as laboratory values, ventilator settings, and sedative requirements. We provide in-depth procedural data particularly pertinent in the COVID-19 pandemic, including number of personnel required per case, apnea time for tracheostomy placement, and both mouth-to-stomach and total procedural time for gastrostomy tube placement, which to our knowledge have not yet been reported in COVID-19 patients. We include 14, 30, and 60-day mortality rates, ICU and hospital lengths of stay, and ultimate disposition in over 98% of patients. Finally, we conducted time-to-event analyses for clinically meaningful prespecified endpoints including time to weaning from mechanical ventilation, tracheostomy downsizing, decannulation, and hospital discharge.

## Methods

### Study Design and Participants

This was a prospective, single-arm, cohort study conducted in the ICU at the Massachusetts General Hospital. Institutional Review Board approval was obtained (#2020P001427). Adult ICU patients who underwent percutaneous tracheostomy with or without percutaneous endoscopic gastrostomy (PEG) for acute respiratory failure due to SARS-CoV-2 were included. Patients who received surgical tracheostomy placement or surgical or radiologic gastrostomy tube placement were excluded.

### Data Collection

Demographic, clinical, procedural, laboratory, radiologic, and outcome data were obtained from the electronic medical record system using a standardized collection process. All data were reviewed by two separate physicians (EF and CO).

### Procedural Technique

Tracheostomies were performed via the percutaneous dilational bronchoscopic-guided technique utilizing a Ciaglia Blue Rhino® kit (Cook Critical Care, Bloomington, IN, USA).^21^ The patient was sedated and received neuromuscular blockade, positioned, and prepped in sterile fashion. All equipment was opened and prepared. A high-efficiency particulate air filter was already in place between the ventilator and tubing. The ventilator was set to volume control at 100% fraction of inspired oxygen (FiO_2_). All personnel other than the primary operators then exited the room and were immediately available outside the glass doors. After careful landmark determination, local anesthetic was injected, the incision made, and the soft tissue bluntly dissected. At this point, in a highly coordinated fashion, the ventilator was paused, and the proximal limb disconnected at the machine. This was done intentionally to reduce aerosolization of viral particles during high risk portions of the procedure. Voluntary apnea was confirmed, and a timer started. The bronchoscope was inserted through the ETT and after rapid ETT cuff deflation, the tube was quickly pulled back to the subglottic space. In a few instances, rapid desaturation was seen. When the oxygen saturation reached 80%, the procedure was interrupted, the ETT was advanced, the circuit closed, and ventilation was briefly resumed. Once preprocedural oxygen saturation was achieved, the circuit was disconnected as described above and the procedure was resumed and finished. Rapid and coordinated Seldinger-technique placement of the tracheostomy tube was carried out, and after cuff inflation, the tracheostomy tube was reconnected to the ventilator circuit. After confirming adequate ventilation and seal of the respiratory system, the team moved to the abdomen.

Gastrostomy tubes were placed via the endoscope-bronchoscope-guided percutaneous method using the 24-French Boston Scientific Pull PEG kit (Boston Scientific Corporation).^17^ Adequate transillumination and digital pressure in the abdomen served as confirmatory measures. The endoscopic equipment used then underwent high-level disinfection as recommended by the manufacturer after each use. These protocols have been proven to have excellent antimicrobial activity.^22,23^

### Statistical Analysis

Standard descriptive statistics were employed using median and interquartile range (IQR) for continuous variables and frequency and percentage for categorical variables. Time to event analyses for prespecified endpoints—weaning from mechanical ventilation, tracheostomy tube downsizing, decannulation, and hospital discharge—–were estimated and plotted using the Kaplan Meier method. We also present the estimated median and time to event as well as 95% confidence interval for these outcomes. Statistical analyses were run using IBM SPSS V25 (Arnonk).

## Results

**Figure 1.**
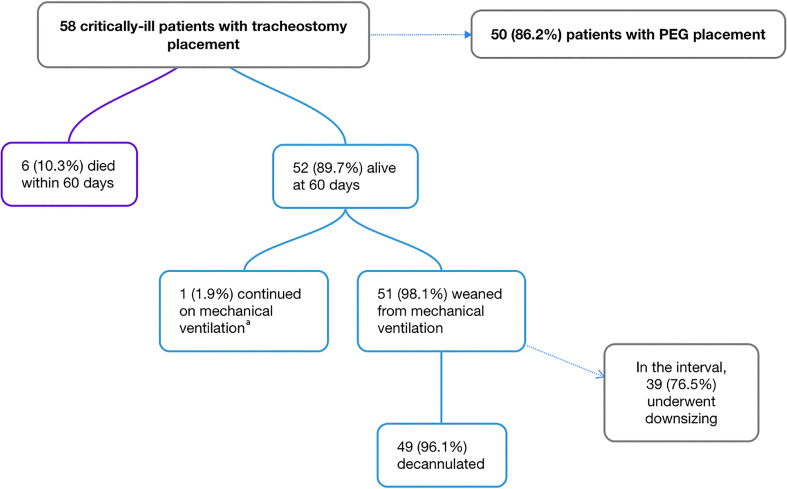
Clinical outcomes in 58 COVID-19 patients with tracheostomy and PEG tube placements. ^a^Patient had preexisting quadriplegia.

Fifty-eight patients were included from April 5, 2020, through June 15, 2020 (Figure 1). Follow-up was extended through August 6, 2020. Demographics and clinical characteristics are shown in Table 1. The median age of patients was 59 years old with a body mass index (BMI) of 27.6. Nearly 90% of the patients received pronation therapy and 5% were either receiving or had received extracorporeal membranous oxygenation (ECMO). An additional 46% of patients underwent ECMO consultation but were found to have an absolute or relative contraindication such as BMI, advanced age, or low likelihood of recovery. All patients were mechanically ventilated with an average positive end-expiratory pressure of 8.0 cm H_2_O and FiO_2_ of 35% the day prior to the procedure. Fifty-six patients were persistently SARS-CoV-2 positive at the time of the procedure.

**Table 1.** Demographic, Clinical, and Laboratory Findings of Cohort.

	Median (IQR) or frequency (%)
	*N* = 58
**Demographics and clinical characteristics**	
Age, years	59 (47.3-66)
Sex	
Women	19 (32.8%)
Men	39 (67.2%)
Height, in	65 (62-67)
Weight, kg	76.2 (68.1-98.2)
BMI	27.6 (24.2-35)
**Comorbidities**	
Malignancy	3 (5.2%)
Immunosuppression	2 (3.4%)
Diabetes mellitus	31 (53.4%)
Chronic kidney disease	12 (20.7%)
Acute kidney injury	38 (65.5%)
Liver dysfunction	18 (31.0%)
**COVID-19 testing**	
Overall % positive	58 (100%)
Persistently positive at time of procedure, %	56 (96.6%)
Days intubated	20 (17-22)
Extubation attempts	0.0 (0-1)
Prior pronation therapy, %	52 (89%)
Evaluated for ECMO, %	30 (51.7%)
Cannulated, %	3 (5.2%)
Contraindication, %	27 (46.5%)
**Medications**	
Propofol	45 (77.6%)
Fentanyl	17 (29.3%)
Midazolam	31 (53.4%)
Ketamine	12 (20.7%)
Dexmedetomidine	23 (39.7%)
Hydromorphone	29 (50.0%)
Antiplatelet agent^a^	5 (8.6%)
Therapeutic anticoagulation^b^	23 (40.3%)
Prophylactic anticoagulation^c^	34 (59.6%)
**Laboratory values**	
White blood cell count, × 10^9^ per L	10.1 (8.3-13.3)
Hemoglobin, g/L	8.5 (7.7-9.5)
Platelet count, × 10^9^ per L	319.5 (206.3-422.8)
International normalized ratio	1.2 (1.1-1.2)
Partial thromboplastin time, s	33 (29.1-39.8)
Blood urea nitrogen, mg/dL	35 (23.8-66)
Creatinine, mg/dL	1.0 (0.6-1.9)
**Imaging (*n* = 39)**	
Normal abdominal X-ray	34 (87.2%)
**Ventilator settings, preprocedure**	
Mode	
Volume control	30 (51.7%)
Pressure control	1 (1.7%)
Pressure support	27 (46.6%)
Positive end expiratory pressure, cmH_2_O	8 (5-8)
Fraction of inspired oxygen, %	35 (30-40)
**Illness severity**	
SOFA score	5 (3-7)

Data are median (IQR) or *n* (%).

Abbreviations: BMI, body mass index; COVID-19, coronavirus disease 2019; SOFA, sequential organ failure assessment; ECMO, extracorporeal membranous oxygenation; IQR, interquartile range.

^a^ Antiplatelet agents include aspirin (325 mg), clopidogrel, prasugrel, ticagrelor.

^b^ Therapeutic anticoagulation defined as treatment dose heparin, enoxaparin, warfarin, apixaban, rivaroxaban.

^c^ Prophylactic anticoagulation defined as prophylactic dose heparin.

Eighty-six percent of patients received both tracheostomy and PEG tube placements with 13.8% receiving tracheostomy alone. Of the 50 patients who had both tracheostomy and PEG tubes placed, 49 were completed in the same procedure during the same anesthetic episode. All procedures were done at bedside with no patient transfer required out of the ICU. A median of 3.0 HCP total were present in the room per procedure. Nursing and respiratory therapists were immediately available outside of the room to provide assistance if needed. Median apnea time during tracheostomy placement was 90 s (IQR 55.8-120). Mouth-to-stomach time for PEG placement was 30 s (IQR 25-53.5) and total PEG procedure time was 14.5 min (IQR 10.6-17). Procedural complications were as follows: minor bleeding in 12 patients, site irritation in six, and repeat procedure needed in five. There were no serious complications such as perforation of adjacent structures or periprocedural death in any patient. Procedural data is summarized in Table 2.

**Table 2.** Procedural Data.

	Median (IQR) or frequency (%)
	*N* = 58
PRBC transfusion prior to procedure, %	11.0 (19.0%)
Units transfused	2.0 (1-6)
Personnel in case	3 (2-3)
Combination tracheostomy and PEG tube placement	
Yes	50 (86.2%)
No	8 (13.8%)
Apnea time, tracheostomy, s	90 (55.8-120)
Time to stomach, PEG, s	30 (25-53.5)
Total procedure time, PEG, min	14.5 (10.6-17)
Procedure-related complications	
Minor bleeding	12 (20.7%)
Site irritation	6 (10.3%)
Need for repeat procedure	5 (8.6%)
Perforation adjacent structure	0 (0%)
Death	0 (0%)

Data are median (IQR) or *n* (%).

Abbreviations: ICU, intensive care unit; PRBC, packed red blood cell; PEG, percutaneous endoscopic gastrostomy; IQR, interquartile range.

**Figure 2.**
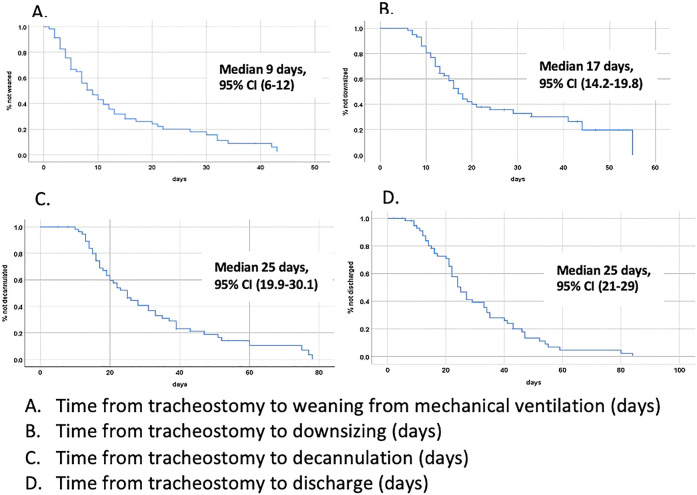
Time to event curves.

Median ICU length of stay (LOS) was 29 days with a median LOS of 10 days postprocedure (see Table 3). Total hospital LOS was 45 days. Nearly 88% of patients overall were weaned from mechanical ventilation postprocedure at a median of 9 days. Ninety-eight percent of patients (*n* = 51) alive at 60 days were successfully weaned. Thirty-nine patients were downsized and 49 were ultimately decannulated. Time to downsizing and decannulation was 17 days and 25 days, respectively (see Figure 2). Mortality rates at 14, 30, and 60 days were 5.2% 6.9%, and 10.3%, respectively. Nearly 90% of patients were discharged alive from the hospital.

**Table 3.** Outcomes.

	Median (95% CI) or frequency (%)
	*N* = 58
Intensive care unit (ICU) length of stay, total, days	29 (24.7-33.3)
Prior to procedure, days	19 (17.4-20.6)
After procedure, days	10 (6.3-13.7)
Hospital length of stay, days	45 (41.9-48.1)
Weaned from ventilator, %	51 (87.9%)
Tracheostomy downsized, %	39 (67.2%)
Decannulated, %	49 (84.5%)
Time from tracheostomy to ventilator weaning, days	9 (6-12)
Time from tracheostomy to downsizing, days	17 (14.2-19.8)
Time from tracheostomy to decannulation, days	25 (19.9-30.1)
Time from tracheostomy to discharge, days	25 (21-29)
Disposition	
Death	6 (10.3%)
Home	5 (8.6%)
Long-term acute care	10 (17.2%)
Rehabilitation unit/center	32 (55.1%)
Skilled nursing facility	5 (8.6%)
Mortality, %	
14 days	3 (5.2%)
30 days	4 (6.9%)
60 days	6 (10.3%)

Data are median (95% CI) or *n* (%).

## Discussion

The COVID-19 pandemic has profoundly impacted patient care worldwide as hospitals and HCP innovate and adapt to the new realities with which they are faced. Critically ill patients with COVID-19 will often need prolonged mechanical ventilation as they recover, raising the question of how this should best be achieved to maximize patient benefit while minimizing risk to HCP. Most medical societies agree that tracheostomy placement should occur between days 10 and 14 of mechanical ventilation, however there is no consensus regarding details such as optimal technique or procedural location. Additionally, minimal data has been published regarding COVID-19 patients and tracheostomy and gastrostomy tube placements. To our knowledge, this is the only study to report clinical outcomes as well as in-depth demographic and periprocedural data in critically ill COVID-19 patients who underwent combined tracheostomy and gastrostomy tube placements.

This study highlights several important points specifically pertaining to tracheostomy and gastrostomy tube placements in COVID-19 patients. First, no transfer out of the patient's room was required for any procedure. This has a profound impact currently as it eliminates exposure to multiple HCP—physicians, nursing staff, respiratory therapists, transport staff, and OR or procedure suite staff. It also decreases contamination and need for terminal cleaning of multiple healthcare areas.

Second, the majority of procedures were performed in the same anesthetic episode, which has multiple benefits. Not only does it decrease the number of times a patient is sedated, it also reduces the exposure to any involved HCP. In this study, these procedures were done with only 2 to 3 HCP per procedure (including combined procedures), without any incidents. This was designed intentionally to expose the least number of HCP to SARS-CoV-2. In follow-up to this study, the physicians primarily involved in performing these procedures tested negative for SARS-CoV-2 IgM and IgG antibodies, indicating they had likely not been exposed despite performing the majority of procedures in actively positive patients. By minimizing the number of HCP involved in these procedures, PPE use was also at a minimum, allowing for conservation of these precious resources. Finally, by using a single endoscope for both procedures, reprocessing need was decreased, thus preventing additional exposure to the staff as well as conserving reprocessing equipment and other cleaning supplies.

Third, our ability to provide nutritional support to patients with critical illness as the disease becomes chronically debilitating is likely to impact their overall outcomes. The published data on gastrostomy tubes and nutrition during the COVID-19 pandemic is very limited so far. A retrospective review involving 6 centers in New York described all cases of endoscopy performed during the COVID-19 pandemic. This included 605 endoscopies in 545 patients with and without COVID-19. Of the 84 endoscopies done on COVID-19 positive patients, nearly one-third of these were performed for PEG or nasogastric tube placement.^24^ This critically ill patient population is likely to be in a hypercatabolic state with increased energy expenditure linked to ventilatory work during their ICU stay. Furthermore, at the time of admission to the hospital, their nutritional status may already be compromised by preexisting factors such as anorexia, dyspnea, anosmia, dysgeusia, confinement, and limited access to food. During their prolonged hospitalization, hypermetabolism and physical immobilization are likely to worsen. The European Society for Clinical Nutrition and Metabolism makes recommendations that favor enteral nutrition over parenteral nutrition, including in the prone position. Nutrition support should be continued after extubation until the patient resumes sufficient oral intake.^25,26^

Fourth, tracheostomy placement in these patients potentially allows for more rapid weaning of sedating medications, which impacts incidence of delirium, weaning from mechanical ventilation, ICU LOS, and development of critical illness polyneuropathy. Multiple trials have demonstrated the importance of daily interruption of sedatives as well as spontaneous awakening and spontaneous breathing trials in mechanically ventilated ICU patients.^27,28^ Tracheostomy has been shown previously to decrease the need for intravenous sedatives and to facilitate patient autonomy earlier compared to continued ventilation via an ETT by increasing comfort, decreasing airway resistance, improving secretion clearance and oral hygiene, and enabling transfer out of the ICU.^10,11,29^ Our data suggests this may be similar in critically ill COVID-19 patients. Additionally, enteral access via the PEG tube allows for administration of medications such as haloperidol or quetiapine which have been shown to decrease agitation in ICU patients, aid with delirium resolution, and increase rates of transfer for patients to home or rehabilitation.^30^

Finally, 19% of patients required preprocedural transfusions and over 40% were on therapeutic anticoagulation that had to be briefly discontinued prior to the procedure(s). In performing combined tracheostomy and gastrostomy tube placements, both the number of transfusions and times anticoagulation was interrupted were potentially decreased by half.

In this study, the 60-day mortality rate was 10.3% (*n* = 6) (Figure 1). This finding is lower than that initially reported in other series of critically ill, mechanically ventilated patients, which was anywhere between 50% and 90%, but is consistent with the trend of decreasing mortality in subsequent reports.^31,32^ While the median sequential organ failure assessment (SOFA) scores in our cohort was only 5, we feel this is not a true reflection of the overall severity of illness. These scores were measured immediately prior to the procedure and are not necessarily the highest overall score for the ICU stay. Additionally, mental status may not have been accurately assessed in the majority of patients due to sedation. This has been described previously as the CNS component of the SOFA score is the least accurate.^33,34^ In a cohort of 59 critically ill COVID-19 patients on mechanical ventilation or mechanical ventilation and ECMO, the average SOFA score was only 6.5.^35^ Nearly 52% of our patients were considered for ECMO and 89% had received pronation therapy for severe respiratory failure, which we feel is a more accurate reflection of the overall severity of illness of the cohort.

Over 98% of patients alive at 60 days had been weaned from mechanical ventilation and 94% of this group had been decannulated. In a cohort of 1890 COVID-19 patients with tracheostomies, 23.7% died by 30-day follow-up. Only 52% of patients were weaned from mechanical ventilation; 81% of those patients were decannulated.^31^ In our cohort, median time to weaning was 9 days, to downsizing was 17 days, and to decannulation was 25 days. Median time to hospital discharge was 25 days. Time to event curves are shown in Figure 2. Prior to tracheostomy and gastrostomy tube placements, patients had a median ICU LOS of 19 days. Postprocedure, median ICU LOS was 10 days. This data supports the use of tracheostomy tubes in COVID-19 patients who require prolonged mechanical ventilation to facilitate weaning and transition out of the ICU to a rehabilitation environment for further recovery. In our cohort, over 80% of our patients required further care at a skilled nursing or long-term acute care facility with excellent overall results.

While bleeding did occur in 20% of patients, this was classified as minor and did not require surgery or embolization. It is important to recognize the coexisting factors in these patients including the frequency of acute renal failure and use of anticoagulation. Fortunately, there were no major complications, such as organ perforation or death, in any patient.

## Conclusion

The COVID-19 pandemic has significantly impacted the delivery of care in critically ill patients. A significant number of these patients will require prolonged mechanical ventilation as they recover from their illnesses. Tracheostomy and gastrostomy tubes are important facilitators of weaning from ventilation and transition out of the ICU into a rehabilitation environment. Here, we report in-depth demographic, clinical, procedural, and outcome data on a single-arm cohort of critically ill patients with COVID-19 who underwent tracheostomy and gastrostomy tube placements. Importantly, nearly 90% of these patients were discharged alive from the hospital and 94% of patients weaned from mechanical ventilation were decannulated. This study supports the use of tracheostomy and gastrostomy tube placement in critically ill COVID-19 patients.
